# Preeclampsia, gestational diabetes and later risk of cardiovascular disease: Women’s experiences and motivation for lifestyle changes explored in focus group interviews

**DOI:** 10.1186/s12884-019-2591-1

**Published:** 2019-11-27

**Authors:** Heidi L. Sandsæter, Julie Horn, Janet W. Rich-Edwards, Hege S. Haugdahl

**Affiliations:** 1 Department of Obstetrics and Gynecology, Levanger Hospital, Helse Nord-Trøndelag Hospital Trust, Postbox 333, 7601 Levanger, Norway; 2 Department of Public Health and Nursing, Faculty of Medicine and Health Sciences, Norwegian University of Science and Technology (NTNU), Postboks 8905, NO-7491 Trondheim, Norway; 3 Department of Epidemiology, Harvard T.H. Chan School of Public Health, Boston, MA USA; 4 Division of Women’s Health, Department of Medicine, Brigham and Women’s Hospital and Harvard Medical School, Boston, MA 02115 USA; 5 Levanger Hospital, Helse Nord-Trøndelag Hospital Trust, Postbox 333, 7601 Levanger, Norway

**Keywords:** Preeclampsia, Gestational diabetes mellitus, Cardiovascular disease, Prevention, women’s health

## Abstract

**Background:**

Preeclampsia (PE) and gestational diabetes mellitus (GDM) are both associated with increased risk of future cardiovascular disease (CVD). Knowledge of the relationship between these pregnancy complications and increased CVD risk enables early prevention through lifestyle changes. This study aimed to explore women’s experiences with PE and/or GDM, and their motivation and need for information and support to achieve lifestyle changes.

**Methods:**

Systematic text condensation was used for thematic analysis of meaning and content of data from five focus group interviews with 17 women with PE and/or GDM, with a live birth between January 2015 and October 2017.

**Results:**

This study provides new knowledge of how women with GDM and/or PE experience pregnancy complications in a Nordic healthcare model. It reveals the support they want and the important motivating factors for lifestyle change. We identified six themes: Trivialization of the diagnosis during pregnancy; Left to themselves to look after their own health; The need to process the shock before making lifestyle changes (severe PE); A desire for information about future disease risk and partner involvement; Practical solutions in a busy life with a little one, and; Healthcare professionals can reinforce the turning point.

The women with GDM wanted healthcare professionals to motivate them to continue the lifestyle changes introduced during pregnancy. Those with severe PE felt a need for individualized care to ensure that they had processed their traumatic labor experiences before making lifestyle changes. Participants wanted their partner to be routinely involved to ensure a joint understanding of the need for lifestyle changes. Motivation for lifestyle changes in pregnancy was linked to early information and seeing concrete results.

**Conclusions:**

Women with PE and GDM have different experiences of diagnosis and treatment, which will affect the follow-up interventions to reduce future CVD risk through lifestyle change. For GDM patients, lifestyle changes in pregnancy should be reinforced and continued postpartum. Women with PE should be informed by their general practitioner after birth, and given a plan for lifestyle change. Those with severe PE will need help in processing the trauma, and stress management should be routinely offered.

## Background

Preeclampsia (PE) is a hypertensive pregnancy complication occurring globally in 2–8% of all pregnancies, which may have serious consequences for both mother and child. Prevalence in Norway is stable at 3–4% [1–3]. Gestational diabetes mellitus (GDM) is increasing globally, with a prevalence of between 1 and 25%, depending on the population group [4]. About 5% of pregnancies in Norway are complicated by GDM [5]. Women who develop PE [6–8] or GDM have a higher risk of cardiovascular disease (CVD) and diabetes mellitus type 2 (T2DM) in later life [9–14]. Preterm birth and/or birth of a growth-retarded baby further increase the risk [11, 15].

In 2011, the American Heart Association included PE and GDM in pregnancy-related risk factors for subsequent development of CVD [15]. Since 2016, European guidelines for prevention and treatment of CVD have indicated PE and GDM as women-specific risk factors to be focused on in monitoring this group of patients [16]. Pregnancies complicated by PE and/or GDM can therefore capture women at increased risk of subsequent development of CVD.

Knowledge of the relationship between these pregnancy complications and increased CVD risk enables early prevention through lifestyle changes. Changes such as increased physical activity, overweight reduction, smoking cessation and healthy diet have been effective in preventing the development of T2DM and CVD [17, 18]. However, a recent systematic review demonstrated that behavior change interventions for diet and physical activity were found to be modestly effective in both the short and long term [19].

Pregnancy and the transition to motherhood can be a period when women are more responsive to suggestions to change their lifestyle. Having responsibility for a baby may also be a motivating factor [20], and may be seen as a teachable moment, increasing the likelihood of implementing a healthier lifestyle [21]. However, for such a teachable moment to lead to health-promoting behavior in pregnant women with PE and/or GDM, they must be familiar with the association with CVD. Life with babies and toddlers is a busy time, when healthy eating habits and physical activity often have low priority [20, 22]. It is therefore crucial that these women can suggest the help and follow-up they need in this change work.

To enhance knowledge of experiences and motivation for lifestyle changes among women with PE and GDM, we suggest drawing on Antonovsky’s salutogenic theory of health promotion and the concept of sense of coherence (SOC) [23]. Confronted with a stressor, the person with a strong SOC will: [1] wish to be motivated to cope (meaningfulness) [2]; believe that the challenge is understood (comprehensibility) [3]; believe that resources to cope are available (manageability). The strength of a person’s SOC is shaped by negative or positive life events and internal (e.g., personality) or external resources (e.g., social support) [24].

Previous US studies have shown that women with PE and/or GDM were unfamiliar with the relationship between GDM and/or PE and increased future CVD risk. GDM and PE were perceived as isolated events in pregnancy without any impact on future health [22]. We lack knowledge about women with PE or GDM facing similar challenges in the Norwegian public health system. Norwegian healthcare is public and free, and antenatal care is provided by GPs and midwives in municipal health services. Postpartum follow-up is mostly performed by GPs.

The study aimed to explore women’s experiences with PE and/or GDM, and their motivation and need for information and support to achieve lifestyle changes. The findings will be used to design a future intervention program for lifestyle changes for these groups.

## Methods

### Participants

Women were eligible for this study if they had a live birth between January 2015 and October 2017 in hospitals under the Nord-Trøndelag Hospital Trust, were at least 18 years old, able to speak and understand Norwegian, and were diagnosed with GDM and/or PE in the hospital database. We confirmed diagnoses of GDM and PE by medical record review, which yielded 67 cases of PE and 103 cases of GDM.

GDM and PE were defined according to current national antenatal guidelines. GDM was defined as onset of glucose intolerance during pregnancy with 75 g 2-h oral glucose tolerance test with fasting glucose ≥5,3–6,9 mmol/l or 2-h value ≥9–11,0 mmol/l [5]. PE complicated by severe hypertension (blood pressure ≥ 160 mmHg systolic or ≥ 110 mmHg diastolic) or with signs of significant end-organ dysfunction was considered severe PE [1].

Potential participants received invitation letters with an information sheet. They reported interest in participation by SMS, and were then telephoned to arrange a date for a focus group interview. They received a reminder by SMS on the interview day to ensure maximum participation.

### Focus group interviews

The focus group interview is a method where a researcher brings together participants who have been selected on the basis of certain common characteristics that relate to the research topic. The researcher creates a relaxing atmosphere to encourage the participants to discuss and share perceptions on a specific topic, with the aim of drawing on the complexity of personal experiences, beliefs, perceptions and attitudes, without any pressure on the participants to vote or reach a consensus [25].

All focus group interviews were conducted between November 2017 and February 2018 in the research department of the hospital and lasted between 90 and 120 min. Participants were 3–34 months post-pregnancy. A semi-structured interview guide (Additional file 1) was used. Before the interview, the participants completed a questionnaire about their demographic characteristics (Additional file 2). The interviews were conducted in diagnosis-specific groups: GDM (three groups, *n* = 10), moderate PE (one group, *n* = 3) and severe PE (one group, *n* = 5). Intra-group homogeneity can enhance group dynamics by increasing the associative effect because of participants’ similar experiences [26].

### Data analysis

The focus group interviews were audio-recorded and transcribed verbatim. Data was analyzed using systematic text condensation (STC), a four-step strategy of cross-case thematic analysis [27]. STC is a modification of Giorgi’s method, further developed by Malterud [28]. In this study, the method was well suited to provide an overview of a large amount of interview data. STC, unlike Giorgi’s method, uses a relatively small number of themes to describe the phenomenon under study, and answers the research question. The filtering process allows the researcher to concentrate further work on the remaining themes [28].

First, focus group interviews were systematically read to gain an overall impression and identify preliminary themes illuminating the research questions. Second, the preliminary themes were organized into code groups and subgroups. Identified meaning units were sorted into these code groups. Third, the meaning units were summarized and developed into artificial quotations or condensates [25]. Forth, the condensates were elaborated into an analytical text.

A codebook with dated reflections validated the analysis. The consolidated criteria for reporting qualitative research (COREQ) were used [29]. Further interpretation of the data was supported by Antonovsky’s salutogenic health theory, centering on essential factors for health promotion [23].

### Ethics

Participants received oral and written information about the study to enable informed choice about participation. They all signed a consent form before the interviews started. The study was approved by the Central Norway Regional Committee for Medical and Health Research Ethics (REK No. 2017/1219) and the Nord-Trøndelag Hospital Trust’s Data Access Committee. During the interviews, participants were informed that their pregnancy complication increased the future risk of CVD and T2DM. They were offered follow-up consultations with an obstetrician (JH) if needed, and two informants with severe PE accepted.

## Results

Seventeen women, mostly living near the hospital, participated in the study. One woman with both GDM and severe PE participated twice. Why some women refused to participate is unknown. The sample showed variation in age, parity, educational level, social background, BMI and urban vs. rural areas. We were unable to recruit non-ethnic Nordic participants (Table 1).

**Table 1:** Descriptive Characteristics of the Study Participants

	Gestational Diabetes Mellitus (*n* = 10)	Moderate Preeclampsia (*n* = 3)	Severe Preeclampsia (*n* = 5)
Maternal characteristics			
Age (years), mean (SD)	33 (59)	30 (5)	35 (4)
Norwegian or other Nordic ethnicity, *n* (%)	10 (100)	3 (100)	5 (100)
Educational level, *n* (%)			
Lower than high school	1 (10)	0 (0)	0 (0)
High school	2 (20)	0 (0)	1 (20)
College or university	7 (70)	3 (100)	3 (80)
Marital status, *n* (%)			
Married	4 (40)	0 (0)	1 (20)
Cohabiting	6 (60)	3 (100)	3 (60)
Single	0 (0)	0 (0)	1 (20)
Occupational status, *n* (%)			
Employed	4 (40)	1 (33)	5 (100)
Employed, maternity leave	4 (40)	2 (67)	0 (0)
Unemployed	2 (20)	0 (0)	0 (0)
Place of residence, *n* (%)			
Urban	4 (40)	1 (33)	1 (20)
Rural	6 (60)	2 (67)	4 (80)
Pre-pregnancy BMI (kg/m^2^), mean (SD)	29 (5)	24(2)	26 (3)
Smoking, *n* (%)	0 (0)	0 (0)	0 (0)
Index pregnancy characteristics			
Nulliparous, (%)	5 (50)	2 (67)	4 (80)
Gestation length (weeks), mean (SD)	38 (3)	38 (2)	34 (3)
Breastfeeding history, *n* (%)			
Full	5 (50)	3 (100)	3 (60)
Partial	4 (40)	0 (0)	1 (20)
No	1 (10)	0 (0)	1 (20)
Delivery mode, *n* (%)			
Vaginal	6 (60)	2 (67)	0 (0)
Elective C-section	2 (20)	1 (33)	0 (0)
Acute C-section	2 (20)	0 (0)	5 (100)

*BMI* Body mass index, *SD* standard deviation

The findings are presented below by six themes and illustrated by quotations from the interviewees.

### Trivialization of the diagnosis during pregnancy

Several participants found that clinicians trivialized their pregnancy complication. Those with GDM stated that clinicians were not always updated on the relevant guidelines for treatment and follow-up care. Some found that despite high values on a glucose tolerance test during pregnancy, they were not referred to follow-up care because their GP mistook the threshold values. Women with severe PE reported delayed referral to specialists, although their blood pressure was above the prescribed level for referral. One woman said this: *“when I was with the midwife I had 140/90, but she sent me home and said it didn’t matter. And I was pleased, because I was going to my sister’s confirmation.”*

Trivialization was a common theme, but differed between the diagnostic groups. Healthcare professionals often stressed that GDM was a short-term and temporary condition.

One woman with insulin-controlled GDM was reassured that one could have sugar in urine and high glucose levels without having GDM. Several had thought that GDM was not very serious, since clinicians did not emphasize information and treatment. One woman with diet-controlled GDM said: *“I was a bit ignorant, I think. I don’t really have diabetes. If so, just a touch. But I probably got too little information for me to take them seriously.”*

All participants with severe PE experienced a serious deterioration of the condition, followed by an emergency delivery. This came as a complete surprise. They had not understood the seriousness of the situation before it was decided to deliver the baby. One woman was told that her high blood pressure was probably due to stress associated with the testing. She had high blood pressure from week 20 until the emergency delivery in week 36. During this time, she had not been referred to a specialist. Another woman was told that her high blood pressure was due to exertion.

### Left to themselves to look after their own health

The participants described different ways of feeling left to themselves, sometimes during, but mostly after, pregnancy. One woman with both GDM and severe PE reported not receiving information or an HbA1c test postpartum as recommended in guidelines [5]. She wished her GP had addressed her pregnancy complications postpartum with information and a plan for follow-up care. Since nobody asked about her GDM, she thought it was not serious. In retrospect, she realized that she should have been reminded about her GDM. Her weight was greater at the interview than at her emergency delivery a year earlier.

Another woman diagnosed with GDM in pregnancy received a letter from her GP with following information: *“You should just change your lifestyle a little.’ ‘Eat healthier food’, it said... That wasn’t much advice. There were only two sentences. But I read up on it myself. I know what to eat and what not to eat. But not everybody knows that ...”*

In particular, the women with diet-controlled GDM felt somewhat alone when having to change their diet to stabilize blood sugar levels. But even those with insulin-controlled GDM found the dietary and exercise advice too superficial. One had only been told to cut out milk. Several with insulin-controlled GDM spoke of inadequate instructions and their fear of injecting themselves with insulin: *“when I got home, my blood sugar level was 12 (mmol/l). An hour later it was 3. That was scary. I felt hungry and then I thought maybe I couldn’t eat because it was so high. But then I thought I had to check it. And then it was very low. It had gone down to 3.”*

One woman who had GDM for the second time felt rebuffed when she contacted the diabetes clinic after giving birth. There was a stark contrast between close outpatient monitoring during pregnancy and a lack of help postpartum. Many participants desired a final meeting with a diabetes nurse, preferably with their partner, focusing on lifestyle advice for the future.

Participants with severe PE had received little follow-up postpartum. Some had not yet recovered from the shock of the diagnosis and their illness experience. One reported not having mentioned her experiences to anyone after discharge. Several were emotionally affected by their experience during the focus group interview. Some cried, and one had written down the whole sequence of events, and partly read this aloud. However, all participants were pleased to share their experiences with similar patients.

### The need to process the shock before making lifestyle changes: severe PE

Participants with severe PE described the trauma of being diagnosed and their subsequent experiences. Several talked about increasing inner turmoil, a vibrating feeling, visual disturbances and intense headaches. They particularly emphasized the experience of being transferred to the intensive care unit (ICU) before the emergency C-section. In the acute phase, they were overwhelmed when the room was suddenly filled up with people starting the treatment. One woman just surrendered and felt that others were in control, while another found it frightening to lose control of what was happening. One woman said: *“I only remember little bits of it all. Like I got magnesium and didn’t tolerate it and … Suddenly I found myself in the ICU and now I’m starting to remember a few more bits after that.”*

Several women still struggled with inner turmoil, which they thought might be related to their continued high blood pressure. Those with severe PE tried hard to get enough rest. Several wanted to learn stress management. Stress was a risk factor they feared would negatively affect their heart. One said:

*I need to calm down instead of going outdoors because I’m struggling with stress. I have to find quiet moments. I have hectic days at work and I’m always busy, so when the kids have gone to bed, I get some calm moments to myself. Yes. So then I don’t go out jogging. That’s not how I want to spend my quiet moments.*

Several participants still had not processed the shock of the diagnosis, the disease experience and their treatment in the most acute phase. They also felt a sense of defeat because their body had been unable to manage a full-term pregnancy, which many believed was natural for the female body. When complications arose, several did not understand the symptoms and therefore sought treatment late. The women had longed for a full-term pregnancy and a normal birth. For some, breast-feeding compensated for “failing” to have a normal full-term birth.

Delayed attachment to the baby was mainly linked to the experience of an emergency delivery and the separation of mother and baby into different wards. One woman felt so little emotional attachment to her child that she thought she would not have noticed it if the staff had given her a different baby. Lack of happiness was another failure. Two participants said that they did not want a second child. Concern about how a new pregnancy would develop led to anxiety about becoming pregnant again. The discussion between the women with severe PE showed that many needed to process their experiences and feeling of failure and learn to manage their stress before making lifestyle changes.

### A desire for information about future disease risk and partner involvement

All participants wanted information about future increased CVD risk, and most considered this vital knowledge. The diagnostic groups had different ideas about the ideal time to receive such information. The women with severe PE wanted to be told by their GP at postpartum checkup, as they needed time to recover, while those with GDM thought the information should be given with the diagnosis but also repeated several times during and after pregnancy. Participants with less severe PE or GDM who felt they already had a healthy lifestyle did not feel that the information was equally important as participants with severe PE, and those without a healthy lifestyle.

Only one woman (who had GDM) was told by clinicians of an increased CVD risk (Table 2). She presumed this was because she had asked many questions during her checkups. Eight women in the GDM group were informed of an increased T2DM risk. The majority of the women in this study reported receiving insufficient information about their pregnancy complication. They wanted clinicians to inform them about causes and symptoms.

**Table 2:** Postpartum Follow-up According to Pregnancy Complication

	Gestational Diabetes Mellitus (*n* = 10)	Moderate Preeclampsia (*n* = 3)	Severe Preeclampsia (*n* = 5)
Received Information on increased CVD risk	1	0	0
Received information on increased T2DM risk	8	0	0
Had a blood pressure measurement	*	2	5
Had a HbA1c measurement	6	*	*

*CVD* Cardiovascular disease, *T2DM* Type 2 diabetes mellitus, *HbA1c* Glycated Hemoglobin Type 1c

*Not asked in focus group interview

Most participants wanted advice and support for lifestyle changes both pre- and postpartum. Those in the GDM group especially desired diet and exercise guidance, and wanted this to start at first diagnosis. They said that clinicians scarcely mentioned lifestyle. The talk with the midwife after birth, meetings with the diabetes nurse and postpartum checkups by their GP and at the clinic were all potential settings for motivation to make healthy lifestyle choices. Only one woman had been recommended to change her lifestyle after giving birth. One participant with GDM stated:

*Someone should have said, ‘I expect you’ve been leading a healthy life now. Try to continue, even though it’s tempting to let yourself go now.’ I’d brought some candy in my labor bag. I’d decided to indulge afterwards. For my part, I think that’s why I haven’t carried on with the changes in my diet.”*

For several, their partner’s diet was a bad influence. Their intentions to eat healthy food were jeopardized by their partner eating unhealthy snacks. One woman with GDM said: *“My partner’s got a very sweet tooth. We’ve tried to discuss eating candy only at weekends and keeping off soft drinks and energy drinks. But when he’s in the store, he’s just got to have some snacks. I’m trying to keep off it but it’s not so easy then.”*

Many participants wished that they and their partner could have been informed together about their pregnancy complications and later CVD risk. This could have underlined the importance of lifestyle changes. One woman felt that talking to a diabetes nurse with her partner after birth was crucial for the family’s decision to take a lifestyle course 8 months postpartum, which improved their lifestyle considerably. The women whose partners supported their need for lifestyle change found that this provided motivation.

### Practical solutions in a busy life with a little one

Many participants had no energy left to exercise and make healthy food. Despite knowing what they ought to do and eat, they did not feel up to it.

One woman described how she and her husband found life challenging on arriving home with their baby. They lived off chocolate and fast food for a long time and hardly got enough sleep. Their baby was premature and only slept for short periods. Another woman explained how her meal and exercise routines fell apart when she became a mother: *“It’s chaos, it’s hectic and I don’t feel I have time for myself. When she’s asleep, I have to lie down and sleep too, I don’t have time to eat. Then sleeping’s more important than eating.”*

Additional life challenges placed extra strain on these mothers. Examples were their own illness, their partner’s or child’s illness, their partner’s commuting or a broken relationship.

Having little time was highlighted as a major barrier to making lifestyle changes. Lack of time led to poorer food choices and less physical activity. Several found they had time but lacked energy. Their main priorities were their child, family, cooking and housework. When evening came, they were exhausted. They concentrated on coping while the children were awake. After the children’s bedtime, all the mothers wanted was the sofa and TV. Sleep and rest had higher priority than physical activity.

Lifestyle changes had to be feasible and adapted to their situation. Most saw diet as most important for risk reduction, emphasizing vegetables and non-processed foods. They agreed that any changes would have to be practicable over time. Everyday physical activity was considered important, but was downplayed. Walks with the stroller and activities with the children were important while they were young. It was necessary to: *“...make sure you have healthy things at home and in the fridge. Don’t buy loads of stuff, because if there’s food in the drawers, at least I can find it when I make coffee. It’s easiest not to have loads of stuff.”*

### Healthcare professionals can reinforce the turning point

One woman with diet-controlled GDM became motivated when she realized the importance of diet for blood sugar and weight. She lost three kilos in 4 weeks by changing her diet. She was determined to continue her new diet to reduce her future risk of T2DM. She said:

*When I saw how important diet was for weight, it was like another kick in the pants. Because I wasn’t very active physically towards the end of my pregnancy. I didn’t have the chance to exercise, so it was really just the diet that did it. I wasn’t aiming to lose weight, but that was like what happened but not consciously. But it doesn’t matter when your BMI’s a bit high.*

One mother of two said she reached a point where she realized she had to do something about her family’s lifestyle. A lifestyle course meant a new start for the whole family. She talked enthusiastically about how this had led to better sleep, a happier mood and more energy. Lifestyle changes were about taking small steps over time. She found her GP to be interested and supportive, which reinforced her motivation to continue.

It was motivating to be told about the relationship between pregnancy complication and an increased future CVD risk. Women with familial accumulation of T2DM or CVD particularly emphasized this as an important motivational factor. One said:

*We were already an active family, but I have to try to do something about my fitness, that’s obvious. But I feel motivated to do that because it runs in the family. Still, it would have been even more motivating if the doctor had looked me in the eyes and said, ‘Hey, this is actually quite important.’ Because I think that’s what lots of people need.*

The participants considered it important to be good health role models, to positively influence their children’s future health and to participate in their children’s activities when they were older. Exercising in groups or with friends could motivate them to start or maintain physical activity.

The participants made many useful suggestions for the content of a lifestyle intervention to reduce the risk of CVD and T2DM to help them maintain a focus on healthy living and prevent them from reverting to bad habits postpartum (Table 3). They wanted the possibility to access the program online at their convenience.

**Table 3:** Participants’ suggestions for the content of an intervention to promote lifestyle changes

Group implementation	
Support groups: Opportunity to meet with women in the same situation.	
Classes in lifestyle change. Dissemination of knowledge. Tips for implementation.	
Stress management classes. Yoga classes.	
Theme of the day/week, e.g. the health promotion effect by drinking water instead of soda.	
Individual implementation	
Kick-off meeting, follow-up meeting, Possibility to chat with a professional, ask questions and get answers.	
Take pictures of meals for quality control. Tips about what to change.	
Suggestions for walking nearby, longer walks at weekends. Exercise with children. Ideas for exercising and increasing everyday activity.	

## Discussion

This study provides new knowledge of how women with GDM and/or PE experience pregnancy complications in a Nordic healthcare model. It reveals the support they want and the important motivating factors for lifestyle change. Our study involved GDM and PE, both of which affect future cardiovascular health, in order to ascertain whether a common approach to these patients was appropriate.

The analysis showed that women with pregnancy complications had difficulty in making necessary lifestyle changes both during and after pregnancy, because they found that healthcare professionals often trivialized their diagnosis. Participants felt left to themselves, especially post-partum. They wished that clinicians were better updated on the diagnosis and understood the importance of well-planned and coordinated treatment and monitoring.

The women with GDM wanted clinicians to motivate them to continue the lifestyle changes introduced during pregnancy. By contrast, those with severe PE felt a need for individualized care to ensure that they had processed their traumatic labor experiences before making lifestyle changes.

Participants generally wanted their partner to be routinely involved to ensure a joint understanding of the need for lifestyle changes. Motivation for lifestyle changes in pregnancy was linked to early information and seeing concrete results. Motivation for long-term lifestyle changes was related to their child’s future health, partner support, practical solutions in a busy life, and healthcare professionals who reinforced the women’s experience of a turning point for lifestyle change.

Women’s experience of trivialization of their pregnancy complication has not previously been described to our knowledge. For the group with GDM, this finding may be due to the introduction of new national guidelines for gestational diabetes in 2017 [5], which involve more comprehensive screening of pregnant women; this is resource-intensive and criticized by some GPs [30].

Women with PE may experience trivialization because clinicians do not want to frighten them by focusing on risks in an already stressful situation. Another explanation may be that Norwegian maternity care considers pregnancy, childbirth and maternity as normal processes in women’s lives [31]. Health professionals’ desire to focus on the normal aspects of pregnancy may have led to less emphasis on the complication.

Many participants in this study felt left to themselves, particularly postpartum. Those with GDM said that professionals in specialist and primary care gave them no encouragement to maintain lifestyle changes introduced during pregnancy. A lack of systematic follow-up made them relapse into bad habits after the birth. This concurs with other studies showing that this group were surprised by the lack of follow-up and felt that their risk of future T2DM and CVD were not addressed postpartum [22, 32–34]. In Norway, different professionals are involved in care for women with pregnancy complications. The desired continuity may be lacking because pregnancy care is mainly at primary level, while specialists are responsible for births and following up complicated pregnancies [35]. Women’s freedom to choose between a GP, midwife or both for pregnancy care leads to poor continuity and consistency of information and care between the various actors in primary and specialist health services [35].

Tierney et al. describe how women found close follow-up at the GDM clinic to be crucial for successful lifestyle changes during pregnancy [34]. Many reverted to previous habits after the follow-up, as described by many women in the present study. Our study confirms that the lack of planned postpartum follow-up of women with PE and/or GDM is unfortunate. They are left to themselves in a vulnerable and often hectic period of maternity; they must combine responsibility for the baby with new routines and maintaining or establishing healthy habits with minimal support. Instead, women wanted to be shown practical solutions to maintain healthy habits during maternity, and to be prepared for how poor sleep and a busy life often encouraged an unhealthy lifestyle. In a salutogenic perspective, being prepared for challenges and seeing possible solutions will increase coping ability [23, 24]. The participants stated that sleep and rest had higher priority than physical activity during this period, which increases the importance of dietary guidance in maternity. The participants’ wishes indicate that a lifestyle intervention for the GDM group should start at diagnosis.

Women with severe PE need to process shock and trauma before starting lifestyle changes. Conditions such as post-traumatic stress disorder (PTSD) or PTSD symptoms double the risk of future CVD [36]. PTSD also has a strong negative effect on a woman’s mental and physical health and her relationship to her child and partner [37]. Women in the severe PE group stated that breastfeeding compensated for their feeling of failure to achieve a normal full-term birth. This is one of several reasons to encourage breastfeeding in this group. Exclusive and partial breastfeeding for 4 months may increase attachment to the baby and perceived mastery, decrease body weight and reduce future CVD risk [38, 39]. A positive breastfeeding experience may thus enhance women’s SOC, which will benefit them in the challenge of changing their lifestyle [23, 24].

Unlike the women in the studies by Seely et al. and Parsons et al., participants in this study did not mention poor finances as a barrier to a healthy lifestyle [22, 40].

Similarly to previous studies, we found that partner support was important for these women in lifestyle change. We are unaware of any knowledge of how partners can promote and maintain lifestyle changes in women with PE and/or GDM. Our participants reported being tempted to copy their partners’ unhealthy eating habits. Many felt that lifestyle change would be facilitated if the whole family realized its importance. In a salutogenic health perspective, the partner is an important resource in encouraging and enabling change [23, 24]. Communication between the couple, the quality of their relationship and the partner’s view of the health risk as a trigger for lifestyle change all influence the goals they set for changing their lifestyle. These factors affect any plans the couple make to address the health risk, and how well the partners’ plans agree [41].

It has little effect merely to provide information on future CVD risk for women with uncomplicated pregnancies and women with pregnancies complicated by severe PE [42]. These women also need practical advice on lifestyle change [42]. A metasynthesis shows that after GDM, women should receive individualized lifestyle change interventions based on their life circumstances [40]. Our participants provided suggestions for the form and content of such an intervention for women with GDM and/or PE (Table 3).

### Study strengths and limitations

Participation in focus groups soon after giving birth was a strength of this study by diminishing the possibility of recall bias. Comparable studies have involved a longer interval between birth and interview [22, 34, 43]. Validation of diagnostic codes enabled the distinction between moderate and severe PE. The focus groups were face-to-face, which could limit distractive elements during the interviews.

Weaknesses of the study are the low recruitment and lack of non-Nordic participants. One focus group contained only two participants due to withdrawals shortly before starting. This is below the recommended group size, but the interview was nevertheless conducted due to the long distance the participants needed to travel. The interview was successful because the conversation between the two women flowed easily and provided new insights into the research questions [25].

## Conclusion

Women with PE and GDM have different experiences of diagnosis and treatment, which will affect the organization of follow-up interventions to reduce future CVD risk through lifestyle change. Women with GDM adapt to their diagnosis through dietary changes and/or insulin therapy, often in the final trimester, while those with PE often experience a brief observation period before induced labor. This difference affects treatment, although some follow-up can be coordinated. For GDM patients, lifestyle changes in pregnancy should be reinforced and continued postpartum. Women with moderate PE have often not changed their lifestyle during pregnancy. They should be routinely informed by their GP after birth, and given a feasible plan for lifestyle change. Those with severe PE will need help in processing the trauma, and stress management should be routinely offered.

## Supplementary information

**Additional file 1.** Interview guide; English language version of the interview guide developed for the focus group study.

**Additional file 2.** Questionnaire; English language version of the questionnaire used to obtain descriptive characteristics.

## Data Availability

The datasets generated and analysed during the current study are not publicly available due to them containing information that could compromise research participants privacy/consent. All data requests should be submitted to the corresponding author for consideration. Access to anonymized data may be granted upon reasonable request given permission of the Central Norway Regional Committee for Medical and Health Research Ethics and the Nord-Trøndelag Hospital Trust’s Data Access Committee.

## Abbreviations

BMI: Body mass index
COREQ: Consolidated criteria for reporting qualitative studies
CVD: Cardiovascular disease
GDM: Gestational diabetes mellitus
GP: General practitioner
ICU: Intensive care unit
PE: Preeclampsia
PTSD: Post-traumatic stress disorder
SOC: Sense of coherence
STC: Systematic text condensation
T2DM: Type 2 diabetes mellitus

## References

[1] Andersgaard AB, Tore H, Langesæter E, Magnussen E, Michhelsen MT, Staff AC (2014). Hypertensive pregnancy complications and Eclampsia [Internett].

[2] Ødegård RA, Vatten LJ, Nilsen ST, Salvesen KÅ, Austgulen R (2000). Risk factors and clinical manifestations of pre-eclampsia. BJOG.

[3] Klungsøyr K, Morken NH, Irgens L, Vollset SE, Skjærven R (2012). Secular trends in the epidemiology of pre-eclampsia throughout 40 years in Norway: prevalence, risk factors and perinatal survival. Paediatr Perinat Epidemiol.

[4] Zhu Y, Zhang C. Prevalence of gestational diabetes and risk of progression to type 2 diabetes: a global perspective. Curr Diab Rep. 2016. 10.1007/s11892-015-0699-x. 10.1007/s11892-015-0699-x PMC6675405 26742932

[5] Helsedirektoratet. Nasjonale faglige retningslinjer for svangerskapsdiabetes (Internett). Oslo: Helsedirektoratet 2017 (updated 25. June 2018; Accessed 26. April 2018). Available from: https://helsedirektoratet.no/retningslinjer/svangerskapsdiabetes

[6] McDonald SD, Malinowski A, Zhou Q, Yusuf S, Devereaux PJ (2008). Cardiovascular sequelae of preeclampsia/eclampsia: a systematic review and meta-analyses. Am Heart J.

[7] Brown MC, Best KE, Pearce MS, Waugh J, Robson SC, Bell R (2013). Cardiovascular disease risk in women with pre-eclampsia: systematic review and meta-analysis. Eur J Epidemiol.

[8] Riise HK, Sulo G, Tell GS, Igland J, Nygard O, Vollset SE, et al. Incident coronary heart disease after preeclampsia: role of reduced fetal growth, preterm delivery, and parity. J Am Heart Assoc. 2017. 10.1161/JAHA.116.004158. 10.1161/JAHA.116.004158 PMC5523993 28264858

[9] Bellamy Leanne, Casas Juan-Pablo, Hingorani Aroon D, Williams David J (2007). Pre-eclampsia and risk of cardiovascular disease and cancer in later life: systematic review and meta-analysis. BMJ.

[10] Sattar Naveed (2004). Do pregnancy complications and CVD share common antecedents?. Atherosclerosis Supplements.

[11] Lykke JA, Langhoff-Roos J, Sibai BM, Funai EF, Triche EW, Paidas MJ (2009). Hypertensive pregnancy disorders and subsequent cardiovascular morbidity and type 2 diabetes mellitus in the mother. Hypertension.

[12] Carr DB, Utzschneider KM, Hull RL, Tong J (2006). Gestational diabetes mellitus increases the risk of cardiovascular diseases in women with a family history of type 2 diabetes. Diabetes Care.

[13] Egeland GM, Skurtveit S, Staff AC, Eide GE, Daltveit AK, Klungsøyr K, et al. Pregnancy-related risk factors are associated with a significant burden of treated hypertension within 10 years of delivery: findings from a population-based norwegian cohort. J Am Heart Assoc. 2018. 10.1161/JAHA.117.008318. 10.1161/JAHA.117.008318 PMC6015329 29755036

[14] Shah BR, Retnakaran R, Booth GL (2008). Increased risk of cardiovascular disease in young women following gestational diabetes mellitus. Diabetes Care.

[15] Mosca L, Benjamin EJ, Berra K, Bezanson JL, Dolor RJ, Lloyd-Jones DM. Effectiveness-based guidelines for the prevention of cardiovascular disease in women-2011 update:a guideline from the american heart association [Internett]. Circulation. 2011; (Accessed 6 June 2018). Available from: https://www.ncbi.nlm.nih.gov/pubmed/21325087. 10.1161/CIR.0b013e31820faaf8 PMC3182143 21325087

[16] Piepoli MF, Hoes AW, Agewall S, Albus C, Brotons C, Catapano AL, et al. 2016 European guidelines on cardiovascular disease prevention in clinical practice (internett) Sophia Antipolis. Eur Heart J. 2016; (Accessed 2 Feb 2018). Available from: https://academic.oup.com/eurheartj/article/37/29/2315/1748952.

[17] Scarborough P, Bhatnagar P, Wickramasinghe K, Rayner M (2011). Trends in coronary heart disease, 1961–2011 (internett).

[18] Aroda VR, Christophi CA, Edelstein SL, Zhang P, Herman WH, Barrett-Connor E (2015). The effect of lifestyle intervention and metformin on preventing or delaying diabetes among women with and without gestational diabetes: the diabetes prevention program outcomes study 10-year follow-up. J Clin Endocrinol Metab.

[19] Samdal GB, Eide GE, Barth T, Williams G, Meland E. Effective behaviour change techniques for physical activity and healthy eating in overweight and obese adults; systematic review and meta-regression analyses. Int J Behav Nutr Phys Act. 2017. 10.1186/s12966-017-0494-y. 10.1186/s12966-017-0494-y PMC5370453 28351367

[20] Edvardsson K, Ivarsson A, Eurenius E, Garvare R, Nyström ME, Small R, et al. Giving offspring a healthy start: parents' experiences of health promotion and lifestyle change during pregnancy and early parenthood. BMC Public Health. 2011. 10.1186/1471-2458-11-936. 10.1186/1471-2458-11-936 PMC3282831 22171644

[21] McBride CM, Emmons KM, Lipkus IM (2003). Understanding the potential of teachable moments for motivating smoking cessation. Health Educ Res.

[22] Seely EW, Rich-Edwards J, Lui J, Nicklas JM, Saxena A, Tsigas E (2013). Risk of future cardiovascular disease in women with prior preeclampsia: a focus group study. BMC Pregnancy Childbirth.

[23] Antonovsky A (1996). The salutogenic model as a theory to guide health promotion. Health Promot Int.

[24] Antonovsky A (2013). Helsens Mysterium, Den salutogene modellen.

[25] Krueger RA, Casey MA (2015). Focus groups: a practical guide for applied research.

[26] Malterud K (2012). Fokusgrupper som forskningsmetode for medisin og helsefag.

[27] Malterud K (2012). Systematic text condensation: a strategy for qualitative analysis. Scand J Public Health.

[28] Malterud K (2017). Kvalitative forskningsmetoder for medisin og helsefag.

[29] Tong A, Sainsbury P, Craig J (2007). Consolidated criteria for reporting qualitative research (COREQ): a 32-item checklist for interviews and focus groups. Int J Qual Health Care.

[30] Backe B (2018). Å skyte spurv med kanoner. Tidskrift for Den norske legeforening.

[31] Helsedirektoratet (2005). Retningslinjer for svangerskapsomsorgen (internett).

[32] Parsons J, Sparrow K, Ismail K, Hunt K, Rogers H, Forbes A. Experiences of gestational diabetes and gestational diabetes care: a focus group and interview study. BMC Pregnancy Childbirth. 2018. 10.1186/s12884-018-1657-9. 10.1186/s12884-018-1657-9 PMC5765597 29325518

[33] Morrison MK, Lowe JM, Collins CE (2014). Australian women's experiences of living with gestational diabetes. Women Birth.

[34] Tierney M, O'Dea A, Danyliv A, Noctor E, McGuire B, Glynn L (2015). Factors influencing lifestyle behaviours during and after a gestational diabetes mellitus pregnancy. Health Psychol Behav Med.

[35] Meld. St. 12 (2008-2009). En gledelig begivenhet: om en sammenhengende svangerskaps-, fødsels- og barselomsorg. Oslo: Helse- og omsorgsdepartement. p. 2009.

[36] Edmondson D, Cohen BE (2013). Posttraumatic stress disorder and cardiovascular disease. Prog Cardiovasc Dis.

[37] Thomson G, Beck C, Ayers S, Thomson G, Schmied V (2017). The rippel effects of a traumatic birth. Risk, impact and implications for practice. Psychosocial resilience and risk in the perinatal period, implications and guidance for professionals.

[38] Kirkegaard H., Bliddal M., Støvring H., Rasmussen K.M., Gunderson E.P., Køber L., Sørensen T.I.A., Nohr E.A. (2018). Breastfeeding and later maternal risk of hypertension and cardiovascular disease – The role of overall and abdominal obesity. Preventive Medicine.

[39] Nguyen Binh, Jin Kai, Ding Ding (2017). Breastfeeding and maternal cardiovascular risk factors and outcomes: A systematic review. PLOS ONE.

[40] Parsons J, Ismail K, Amiel S, Forbes A (2014). Perceptions among women with gestational diabetes. Qual Health Res.

[41] Lewis MA, McBride CM, Pollak KI, Puleo E, Butterfield RM, Emmons KM (2006). Understanding health behavior change among couples: an interdependence and communal coping approach. Soc Sci Med.

[42] Bokslag A, Kroeze W, de Groot CJM, Teunissen PW (2018). Cardiovascular risk after preeclampsia: the effect of communicating risk factors on intended healthy behavior. Hypertens Pregnancy.

[43] Razee H, van der Ploeg HP, Blignault I, Smith BJ, Bauman AE, McLean M (2010). Beliefs, barriers, social support, and environmental influences related to diabetes risk behaviours among women with a history of gestational diabetes. Health Promot J Austr.

