# Supplementary material for: Preeclampsia, gestational diabetes and later risk of cardiovascular disease: Women’s experiences and motivation for lifestyle changes explored in focus group interviews
Source: BMC Pregnancy Childbirth. 2019 Nov 27;19:448. doi: 10.1186/s12884-019-2591-1 (PMC6882194; doi:10.1186/s12884-019-2591-1)
Supplement: Supplementary file 1 — Additional file 1. Interview guide; English language version of the interview guide developed for the focus group study. [file 12884_2019_2591_MOESM1_ESM.docx]

**Interview Guide**

Information

- Start by mentioning that the moderator and secretary are legally bound to secrecy. We would also like you, the participants, to agree on confidentiality within this group.
- It is up to each individual person how much sensitive information you want to share. Everything said here will be anonymized after the data has been processed. All the material will be stored in a locked cabinet.
- The focus group interview will take about 90 minutes
- There are no right or wrong answers. We would like you all to contribute to the discussion.
- We are interested in your experiences, not so much general comments. We hope you can give some specific examples. The interview can very well be a conversation between all of you; I will elaborate, follow up and sometimes introduce new topics.

1. Please tell us about your experience of PE/GDM. Let us know when you were diagnosed and what happened after that.

1. Did anyone talk to you about the connection between PE/GDM and disease in later life?
2. Who gave you this information?

**Inform the participants about PE/GDM and future increased risk of CVD and T2DM.**

1. How did you feel about getting this information?
2. Is it important to get this information?
3. When would have been the best time to get this information?

**Inform the participants that there is a good chance of preventing future CVD. It is an advantage that their risk was identified at an early stage. Diet, exercise, quitting smoking and monitoring of blood pressure and blood sugar are the most important preventive measures**.

1. Now that you are aware that PE/GDM increases the risk of future CVD, how does this affect your motivation to make lifestyle changes to reduce future risk?
2. What motivates you the most to make lifestyle changes?
3. What does the idea of lifestyle changes mean to you? What is activity? What is a healthy diet?
4. What do you find prevents you from making lifestyle changes?
5. What is the biggest barrier?
6. What assistance and support/follow-up do you need from the health service and those around you to make lifestyle changes?
7. Researchers at the Norwegian University of Science and Technology are developing an app to promote lifestyle changes after pregnancy complicated by PE/GDM. This app will be available for mobile devices or computers. Professionals will support the service by providing individual advice and follow-up.
8. What do you think this app should include?
