# Supplementary material for: Preeclampsia, gestational diabetes and later risk of cardiovascular disease: Women’s experiences and motivation for lifestyle changes explored in focus group interviews
Source: BMC Pregnancy Childbirth. 2019 Nov 27;19:448. doi: 10.1186/s12884-019-2591-1 (PMC6882194; doi:10.1186/s12884-019-2591-1)
Supplement: Supplementary file 2 — Additional file 2. Questionnaire; English language version of the questionnaire used to obtain descriptive characteristics. [file 12884_2019_2591_MOESM2_ESM.docx]

# questionnaire **-** descriptive characteristics

**First name**:

**Age**:

**Place of residence**:

Urban Rural

**Marital status**:

 and the Nord-Trøndelag Hospital Trust’s Data Access Committee and the Nord-Trøndelag Hospital Trust’s Data Access Committee and the Nord-Trøndelag Hospital Trust’s Data Access Committee

Married Single Cohabitant

**Educational level:**

Lower than high school High school College or university

**Occupational status:**

Employed Employed, maternity leave Unemployed

**Delivery mode:**

Vaginal Elective C-section Acute C-section

**Gestation length at delivery (weeks):**

**Breastfeeding history:**

:

Full Partial No

**Parity:**

Primiparous Multiparou**s**

**Smoking:**

Yes No

**Free text for additional information**
